# Complement Alternative Pathway Activation in Human Nonalcoholic Steatohepatitis

**DOI:** 10.1371/journal.pone.0110053

**Published:** 2014-10-09

**Authors:** Filip M. Segers, Froukje J. Verdam, Charlotte de Jonge, Bas Boonen, Ann Driessen, Ronit Shiri-Sverdlov, Nicole D. Bouvy, Jan Willem M. Greve, Wim A. Buurman, Sander S. Rensen

**Affiliations:** 1 Department of General Surgery, Maastricht University Medical Centre+, Maastricht, the Netherlands; 2 Department of Surgery, Atrium Medical Centre Parkstad, Heerlen, the Netherlands; 3 Department of Pathology, Maastricht University Medical Centre+, Maastricht, the Netherlands; 4 Department of Genetics and Cell Biology, Maastricht University Medical Centre+, Maastricht, the Netherlands; University of Basque Country, Spain

## Abstract

The innate immune system plays a major role in the pathogenesis of nonalcoholic steatohepatitis (NASH). Recently we reported complement activation in human NASH. However, it remained unclear whether the alternative pathway of complement, which amplifies C3 activation and which is frequently associated with pathological complement activation leading to disease, was involved. Here, alternative pathway components were investigated in liver biopsies of obese subjects with healthy livers (n = 10) or with NASH (n = 12) using quantitative PCR, Western blotting, and immunofluorescence staining. Properdin accumulated in areas where neutrophils surrounded steatotic hepatocytes, and colocalized with the C3 activation product C3c. C3 activation status as expressed by the C3c/native C3 ratio was 2.6-fold higher (p<0.01) in subjects with NASH despite reduced native C3 concentrations (0.94±0.12 vs. 0.57±0.09; p<0.01). Hepatic properdin levels positively correlated with levels of C3c (r_s_ = 0.69; p<0.05) and C3c/C3 activation ratio (r_s_ = 0.59; p<0.05). C3c, C3 activation status (C3c/C3 ratio) and properdin levels increased with higher lobular inflammation scores as determined according to the Kleiner classification (C3c: p<0.01, C3c/C3 ratio: p<0.05, properdin: p<0.05). Hepatic mRNA expression of factor B and factor D did not differ between subjects with healthy livers and subjects with NASH (factor B: 1.00±0.19 vs. 0.71±0.07, p = 0.26; factor D: 1.00±0.21 vs. 0.66±0.14, p = 0.29;). Hepatic mRNA and protein levels of Decay Accelerating Factor tended to be increased in subjects with NASH (mRNA: 1.00±0.14 vs. 2.37±0.72; p = 0.22; protein: 0.51±0.11 vs. 1.97±0.67; p = 0.28). In contrast, factor H mRNA was downregulated in patients with NASH (1.00±0.09 vs. 0.71±0.06; p<0.05) and a similar trend was observed with hepatic protein levels (1.12±0.16 vs. 0.78±0.07; p = 0.08). Collectively, these data suggest a role for alternative pathway activation in driving hepatic inflammation in NASH. Therefore, alternative pathway factors may be considered attractive targets for treating NASH by inhibiting complement activation.

## Introduction

In recent decades, the incidence and prevalence of nonalcoholic fatty liver disease (NAFLD) has dramatically increased [1]. NAFLD can progress from relatively benign hepatic fat accumulation or steatosis to more severe stages characterized by hepatic inflammation, in a condition referred to as nonalcoholic steatohepatitis (NASH). NASH, in turn, may lead to fibrosis, cirrhosis, liver failure, and even hepatocellular carcinoma [2]. In spite of the high prevalence of NAFLD, its etiology and the mechanisms responsible for progression towards nonalcoholic steatohepatitis (NASH) remain to be fully elucidated [2], [3].

Complement activation is classically considered an important antimicrobial defense system. However, accumulating evidence associates complement activation with inflammatory conditions such as transplant rejection, neurodegenerative diseases, ischemia/reperfusion damage, and cancer. Additionally, key functions of complement in immune surveillance, homeostasis, and mediation of inflammatory responses have been elucidated. Complement factors not only sense and eliminate foreign pathogens, but also target altered-self, diseased, and apoptotic cells. Therefore, excessive activation or dysregulation of the complement system may have far-reaching clinical consequences [4].

Complement activation can be initiated through three different pathways, i.e. the classical pathway, the lectin pathway, and the alternative pathway. Previously, we have shown that the classical and lectin branches of the complement system are involved in the progression of NAFLD in a significant proportion of patients [5]. NAFLD severity was associated with accumulation of activation products of C3, the central complement component, around steatotic hepatocytes. Several components of the classical and lectin pathways, including C1q, MBL, and C4d, were also found to accumulate in the liver of subjects with NAFLD. However, C3 activation was not accompanied by C1q, MBL, or C4d deposition in all patients, suggesting that the alternative pathway could also be involved in complement activation in NAFLD.

The alternative pathway is unique compared with the other two pathways because it provides a potent positive feedback loop, amplifying the activation of C3 irrespective of the pathway responsible for the initial activation. Activation of the alternative pathway thus strongly increases the production of all complement-related pro-inflammatory effectors. Indeed, it is the alternative pathway that appears to drive pathological complement activation that leads to disease [6].

Alternative pathway activation is critically dependent on properdin, a positive regulator of the assembly of C3bBb, the alternative pathway C3 convertase [7]. Properdin is stored in neutrophils and can be rapidly released upon their activation [8]. Given the very low plasma properdin levels [9], local release of properdin from neutrophils is considered the major determinant of alternative pathway activity [10]. Importantly, neutrophils have already been shown to make a major contribution to the inflammatory process in NASH [11], [12]. Moreover, complement activation in subjects with NASH appears to be associated with hepatic infiltration of neutrophils near steatotic hepatocytes [5], [11].

In view of 1) the fact that complement activation in NASH can only be partially attributed to classical and lectin pathway induction, and 2) the accumulation of properdin-bearing neutrophils around steatotic hepatocytes, we hypothesized that locally induced alternative pathway activation could be important for driving complement activation in NASH. Here, we show for the first time that hepatic properdin levels are related to both C3 activation and the extent of lobular inflammation in human NASH, suggesting that the alternative pathway plays an important role in complement activation in NASH. Since therapeutic inhibition of the complement cascade can and should be directed to specific pathways to limit side effects [6], this knowledge is crucial for enabling the development of novel treatment strategies for NAFLD based on complement inhibition.

## Methods

### Subjects, biopsies, and NASH severity

Liver wedge biopsies were obtained from subjects undergoing bariatric surgery at the Maastricht University Medical Centre (Table 1). None of the subjects reported alcohol intake>20g/day or suffered from autoimmune diseases or viral hepatitis. This study was approved by the local Medical Ethics Committee of Maastricht University Medical Centre, according to the revised version of the Declaration of Helsinki (October 2008, Seoul). The principles of good clinical practice (GCP) were followed during this study. Written informed consent was obtained from every patient before study participation.

**Table 1:** Patient characteristics.

	Control	NASH	p-value
Sex (Female/Male)	9/1	9/3	0.59
Age (years)	41.1±2.6	45.0±2.7	0.35
BMI (kg/m^2^)	44.2±2.0	53.8±4.0	0.12
Fasting glucose (mmol/l)	5.6±0.2	7.7±0.8	0.06
ALT (IU)	18.8±2.1	37.7±5.3	<0.01
AST (IU)	17.1±2.0	30.6±3.3	<0.01
NAS score	0±0	8.0±0.5	<0.01

BMI – Body Mass Index; Data are shown as mean ± SEM.

Liver biopsies were snap-frozen in liquid nitrogen for mRNA/protein analysis, or fixed in 4% formalin and embedded in paraffin for immunohistochemistry.

NASH severity was assessed by an independent liver pathologist according to the NAFLD Activity Score (NAS score) defined by Kleiner[13] and the Brunt classification [14]. Two groups of subjects with established NASH (NAS score≥6; N = 12) or without NASH (NAS score = 0; N = 10) were compared (Table S1).

### Quantitative polymerase chain reaction

RNA extraction was performed with TRI-reagent (Sigma-Aldrich, St. Louis, MO) according to the manufacturer's protocol. 750 ng RNA was used for reverse transcription using the iScript cDNA synthesis kit (Bio-Rad, Hercules, CA). Quantitative polymerase chain reaction (qPCR) reactions were performed in a volume of 20µL consisting of 10 ng cDNA, 1× Absolute qPCR SensiMix (GC Biotech, Alphen aan de Rijn, the Netherlands), and 150 nM of gene-specific primers (Table S2). Complementary DNA was amplified using a 3-step program (40 cycles of 10 seconds at 95°C, 20 seconds at 60°C, and 20 seconds at 70°C) with a MyiQ system (Bio-Rad). Specificity of amplification was verified by melt curve analysis. Gene expression levels were determined with iQ5 software using the ΔCt relative quantification model. The geometric mean of 2 internal control genes cyclophilin A and beta2-microglobulin was calculated and used as normalization factor.

### Western blotting

Liver tissue was homogenized in RIPA lysis buffer (50 mM Tris buffer, 150 mM NaCl, 10% glycerol, 1% Nonidet P-40 and 0,1% SDS) using glass beads in a Mini-Beadbeater (Biospec, Bartlesville, OK). After centrifugation (18000 g for 15 min. at 4°C), protein concentration was determined using a BCA protein assay kit (Pierce Thermo Fisher Scientific Inc., Rockford, IL). Proteins (10µg) were heated (95°C) in a sodium dodecyl sulfate sample buffer containing beta-mercaptoethanol, loaded on a 12% sodium dodecyl sulfate polyacrylamide gel and blotted onto a polyvinylidenefluoride membrane (Immobilon P, Millipore, Bedford, MA). Membranes were incubated with monoclonal anti-C3c (1∶1000, Dako, Glostrup Denmark), polyclonal anti-properdin (1∶500, Nordic Immunology, Tilburg, the Netherlands), polyclonal anti-factor H (1∶500, Abcam ab8842, Cambridge, UK) or monoclonal anti-DAF antibodies (1∶500, kindly provided by Dr. D. Lublin, St. Louis, MO) overnight at 4°C. After thorough washing with TBS, membranes were incubated with appropriate HRP-conjugated secondary antibody for 1.5 h at room temperature. To ensure equal loading and transfer, membranes were reprobed with mouse anti–beta-actin (Sigma) and rat anti-mouse horseradish peroxidase–conjugated antibody (Jackson ImmunoResearch Laboratories) was used as secondary antibody. Signal was detected using the chemiluminescent substrate Supersignal West Pico (Pierce Thermo Fisher Scientific Inc.) on X-ray film (SuperRX, Fuji, Tokyo, Japan). Band intensity was analyzed and quantified using Quantity One (Bio-Rad) and corrected for protein loading using the beta-actin band intensity.

### Immunohistochemistry

For immunofluorescent double staining of MPO/properdin and C3c/properdin, 4µm thick liver sections were rehydrated and antigen binding sites were retrieved using preheated (95°C) Antigen Retrieval Buffer (Dako) for 30′, followed by a cooling down period of 20′. Non-specific binding sites were blocked with 10% goat serum in PBS. Sections were incubated overnight with rabbit-anti-MPO (Dako; 1∶1000) and monoclonal anti-C3c (1∶200) or goat-anti-properdin (Nordic Immunology, 1∶250) and monoclonal anti-C3c (1∶200) at 4°C, rinsed with PBS, and subsequently incubated with Alexa Fluor 488-conjugated donkey anti-goat (Invitrogen Molecular Probes, Eugene, OR, 4µg/ml) for 1,5 h at room temperature, followed by an appropriate Cy3-conjugated IgG antibody (Invitrogen, 1∶500) for 1,5 h at room temperature. Nuclei were stained with 4′,6-diamino-2-phenyl-indol (DAPI), and sections were mounted with Fluorescent Mounting Medium (Dako) and observed with a Leica immunofluorescence microscope.

### Statistical Analysis

Results are presented as mean±SEM in the manuscript. Graphs are presented as Tukey box and whiskers, with whiskers from min to max and the line representing the median value; mean values are indicated with ‘+’. Statistical analyses were conducted using Graphpad Prism 5.02 software (Graphpad, San Diego, CA). ANOVA and Mann Whitney test were applied to analyze differences among the study groups. Associations between parameters were determined by Spearman correlation analysis. P-values <0.05 were considered statistically significant and are indicated as follows in the graphs: * p<0,05, ** p<0,01 and *** p<0,001.

## Results

### Alternative pathway activation in NASH

Previously, we have reported increased activation of C3, the central complement protein, in the liver of patients with NASH, frequently in parallel with accumulation of both classical and lectin pathway components. However, not all C3 activation could be associated to the classical or lectin pathways, suggesting an additional role for the alternative pathway. Since properdin is a pivotal positive regulator of the alternative pathway by stabilizing alternative pathway convertases, we first assessed whether it accumulated in the liver of subjects with NASH.

**Figure 1:**
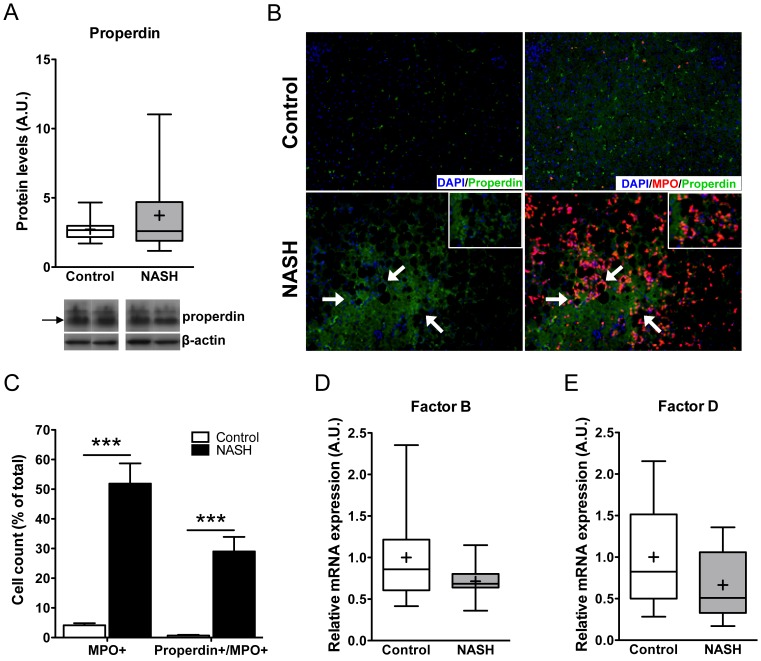
Alternative pathway related factors in NASH. A) Hepatic properdin protein levels were not significantly different between subjects with a healthy liver and subjects with NASH (p = 0.92). B) Representative images of immunofluorescent stainings for properdin (green) and myeloperoxidase (MPO, red), showing pronounced extracellular accumulation of properdin in areas where neutrophils surround steatotic hepatocytes in subjects with NASH (100× magnification), whereas control subjects with healthy livers display no or little properdin accumulation and neutrophil infiltration (100× magnification). C) Quantitative analysis of immunofluorescent staining for MPO+ cells (***p<0.001) and properdin+/MPO+ cells (***p<0.001) in healthy livers and livers from subjects with NASH. D) Hepatic factor B mRNA expression was comparable in subjects with healthy livers and subjects with NASH (p = 0.26). E) Similar mRNA expression of factor D in the study groups (p = 0.29).

Subjects with NASH displayed hepatic properdin protein levels that were comparable to control subjects with healthy livers (3.73±0.83 vs. 2.72±0.25; p = 0.92, figure 1A). However, immunofluorescent staining for properdin and the neutrophil marker myeloperoxidase revealed a strong extracellular accumulation of properdin in areas where neutrophils surrounded steatotic hepatocytes (figure 1B, lower panel). No or little extracellular properdin accumulation was observed in subjects with healthy livers (figure 1B, upper panel). Quantification of the immunofluorescent images for MPO+ and properdin+/MPO+ double positive cells showed both significant increases in infiltrated MPO+ neutrophils (51.90±6.79% vs. 4.15±0.70%; p<0.001) and properdin+/MPO+ double positive cells (29.04±4.89% vs. 0.66±0.24% of MPO+ cells; p<0.001) in subjects with NASH compared to healthy controls (figure 1C).

The main function of properdin, as a positive regulator of the alternative pathway, is stabilization of the alternative pathway convertase C3bBb. Since factor B and factor D are necessary for the formation of C3bBb, we next analyzed their expression. Factor B and factor D levels were not significantly different between subjects with healthy livers and subjects with NASH (factor B: 1.00±0.19 vs. 0.71±0.07; p = 0.26; factor D: 1.00±0.21 vs. 0.66±0.14; p = 0.29, figure 1C and 1D). These data suggest that whereas regulatory components of the alternative pathway are not differentially expressed in human NASH, neutrophils surrounding steatotic hepatocytes may still serve as an important source of properdin.

### C3 activation in human NASH correlates with hepatic properdin levels

Since the local accumulation of properdin in subjects with NASH could drive alternative pathway mediated activation of C3, we next investigated whether hepatic properdin levels were associated with C3 activation.

**Figure 2:**
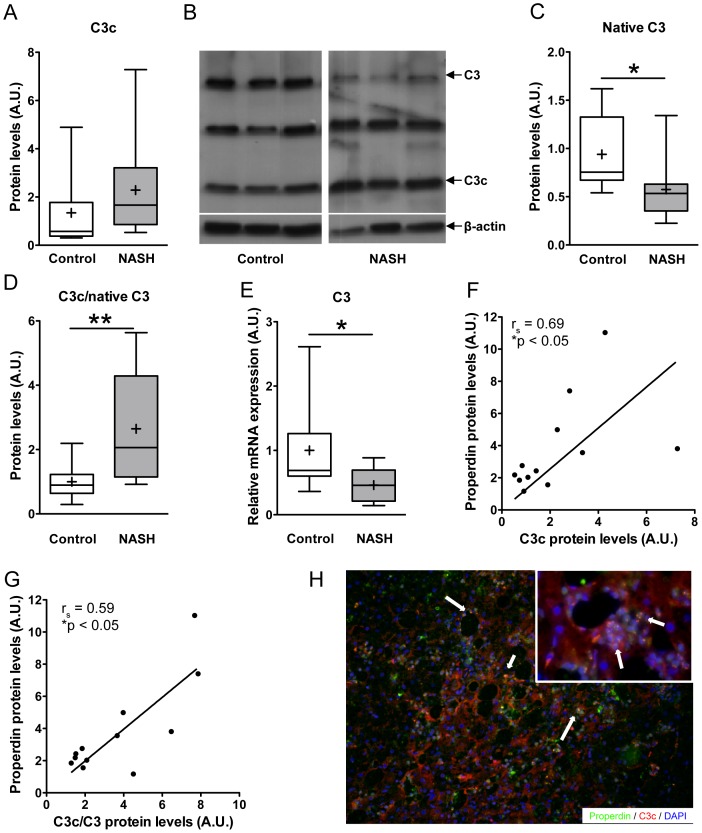
C3 activation in human NASH. A) Semi-quantitative analysis of Western blot (see 2B) for hepatic protein C3c levels in subjects with NASH and subjects with healthy livers (p = 0.24). B) Western blot showing a clear shift from the native C3 band towards the smaller C3c band in subjects with NASH (see arrows). In contrast, healthy subjects showed high concentrations of native C3 and low levels of C3c (see arrows). C) Semi-quantitative analysis of Western blots confirmed significantly lower levels of native C3 protein in the liver of subjects with NASH (*p<0.05). D) The ratio of C3c/native C3, reflecting the activation status of C3, was higher in subjects with NASH compared to controls (**p<0.01). E) Decreased hepatic C3 mRNA expression in subjects with NASH compared to control subjects (*p<0.05). F) In subjects with NASH, hepatic properdin protein expression levels correlated significantly with C3c protein levels (r_s_ = 0.69; *p<0.05). G) A similar correlation in subjects with NASH was observed between properdin and the C3c/native C3 ratio (r_s_ = 0.59; *p<0.05). H) Representative image of immunofluorescent stainings for properdin (green) and C3c (red), showing pronounced colocalization of properdin and C3 activation (C3c) in areas with steatotic hepatocytes in subjects with NASH.

First, we assessed C3 activation in relation to NASH. Semi-quantitative analysis of hepatic C3c protein levels by Western blotting showed a trend towards an increase in C3c protein levels in subjects with NASH as compared with subjects with healthy livers (2.29±0.57 vs. 1.35±0.50; p = 0.07; figure 2A). Because the C3c antibody we used also recognizes the alpha-chain of the native C3 protein, a clear shift on Western blot could be observed from the native C3 band towards the smaller C3c band in subjects with NASH (figure 2B). Subjects with healthy livers showed high concentrations of native C3 and low levels of the cleaved/activated C3 protein, whereas subjects with NASH showed high levels of C3c and low levels of native C3 proteins (figure 2B). In fact, native C3 protein levels were significantly lower in subjects with NASH (0.94±0.12 vs. 0.57±0.09; p<0.05; figure 2C). The ratio of C3c/native C3 was used as a marker of the activation status of C3. Interestingly, subjects with NASH showed a significant increase in the C3c/native C3 activation ratio compared to controls with healthy livers (2.65±0.51 vs. 1.00±0.17; p<0.01; figure 2D). In line with the native C3 protein data, qPCR analysis showed a significant>2-fold decreased C3 mRNA expression in subjects with NASH compared to control subjects (1.00±0.23 vs. 0.46±0.09; p<0.05; figure 2E). Importantly, a strong correlation was observed between hepatic C3c and properdin protein levels in subjects with NASH (r_s_ = 0.69; p<0.05; figure 2F), whereas this correlation was absent in subjects with healthy livers (r_s_ = −0.27; p = 0.40; data not shown). A similar significant correlation was also observed between hepatic properdin protein levels and the C3c/native C3 activation ratio (r_s_ = 0.59; p<0.05; figure 2G). Furthermore, immunofluorescent staining of liver tissue of patients with NASH showed colocalization of C3c and properdin in the crown-like structures surrounding steatotic hepatocytes (figure 2H). These data support that C3 activation in human NASH is related to alternative pathway activation, and show that hepatic C3 levels are reduced in NASH while the C3 present is mostly activated.

### Alternative pathway inhibitor levels in human NASH

Activation of the alternative complement pathway is regulated at multiple levels. Factor H, a soluble/secreted protein primarily synthesized by the liver, plays a particularly important role in controlling alternative pathway activation by inhibiting formation of the C3 convertase C3bBb and by accelerating its degradation. In addition factor I, a plasma serine protease, plays an important role in inhibiting the alternative pathway amplification loop by cleaving C3b to inactive iC3b.

**Figure 3:**
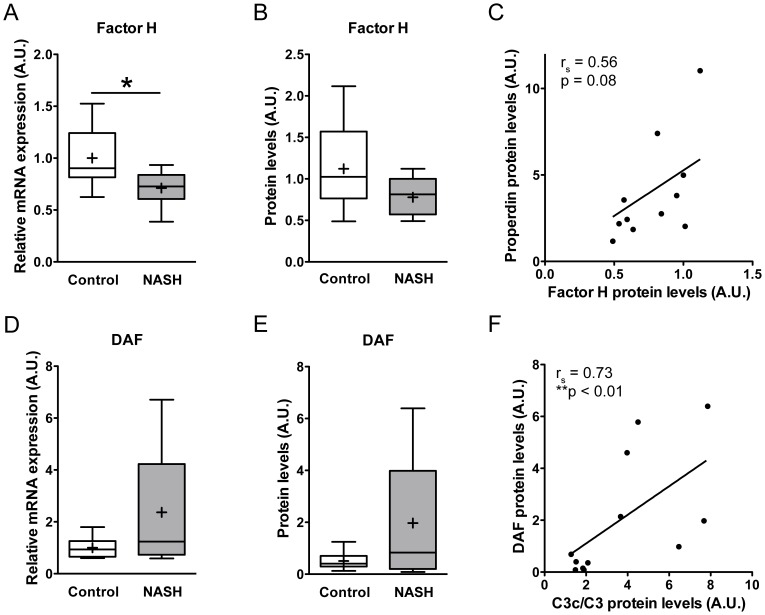
Alterations in regulators of the alternative pathway in human NASH. A) Reduced factor H mRNA expression in the liver of subjects with NASH compared to subjects with healthy livers (*p<0.05). B) Semi-quantitative analysis of hepatic factor H protein levels (p = 0.08). C) Correlation between hepatic factor H and hepatic properdin protein levels in subjects with NASH (r_s_ = 0.56; p = 0.08). D) Hepatic DAF mRNA expression in patients with NASH compared to controls (p = 0.22). E) The increase in hepatic DAF protein levels in subjects with NASH was not statistically significant (p = 0.28). F) However, there was a significant correlation between hepatic DAF protein levels and the activation ratio of C3c/native C3 in subjects with NASH (r_s_ = 0.73; **p<0.01).

Interestingly, subjects with NASH showed a significantly lower hepatic mRNA expression of factor H compared to subjects with healthy livers (0.71±0.06 vs. 1.00±0.09; p<0.05; figure 3A). Semi-quantitative Western blot analysis of hepatic factor H protein levels showed a similar trend towards decreased factor H protein levels in subjects with NASH compared to control subjects (0.78±0.07 vs. 1.12±0.16; p = 0.08; figure 3B). Although hepatic factor H protein levels did not show strong correlations with hepatic C3c protein levels (r_s_ = 0.46, p = 0.15, data not shown) or C3 activation (C3c/native C3 ratio) (r_s_ = 0.36, p = 0.27, data not shown), hepatic factor H protein levels tended to correlate with hepatic properdin protein levels (r_s_ = 0.56, p = 0.08, figure 3C).

Next to factor H, the membrane-bound glycoprotein DAF has an important protective role in the regulation of the complement cascade by accelerating the decay of alternative and classic C3-convertases. Hepatic DAF mRNA and protein levels showed a trend to be increased in patients with NASH compared to controls (mRNA: 2.37±0.72 vs. 1.00±0.14; p = 0.22, figure 3D/Protein: 1.97±0.67 vs. 1.00±0.21; p = 0.28, figure 3E). Hepatic protein levels of DAF were not correlated with C3c protein levels in subjects with NASH (r_s_ = 0.49, p = 0.11, data not shown). However, when looking at C3 activation expressed as the ratio of C3c/native C3, a clear and significant correlation was observed with hepatic DAF protein expression in NASH (r_s_ = 0.73, p<0.01, figure 3F).

### Association between hepatic properdin, DAF, and C3c with steatosis, lobular inflammation, and NASH severity

**Figure 4:**
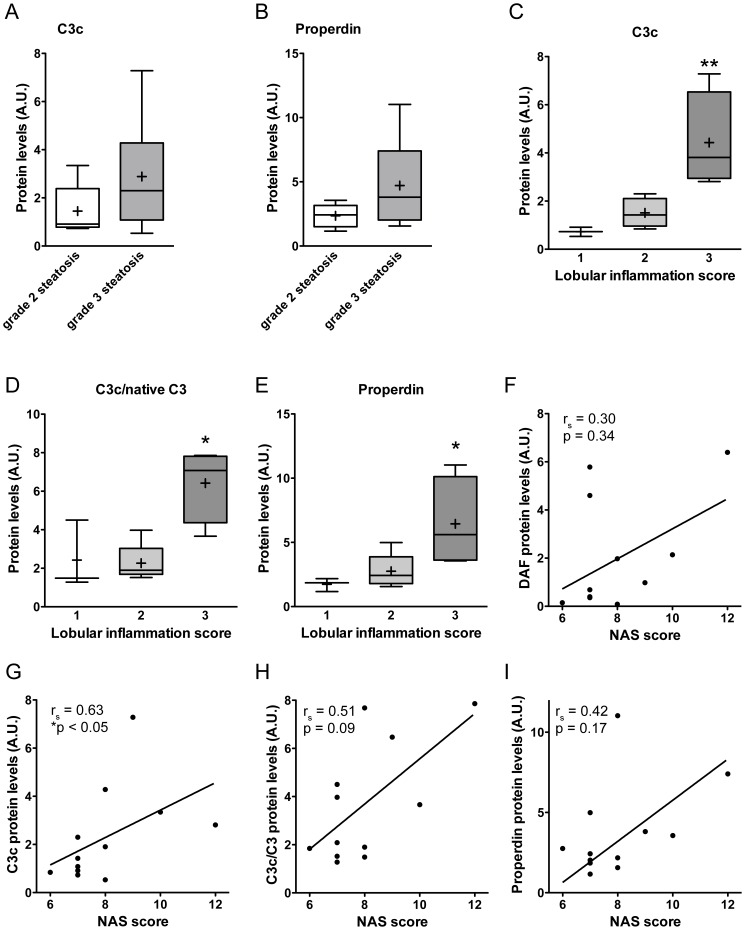
Association between properdin, DAF, and C3c with hepatic steatosis and inflammation. A) C3c levels in the liver were not different (p = 0.27) between subjects with grade 2 steatosis (N = 5) and subjects with grade 3 steatosis (N = 7). B) Levels of hepatic properdin were not related to the grade of steatosis (p = 0.27). C) Progressively increasing levels of hepatic C3c with higher lobular inflammation scores (**p<0.01). D) Hepatic C3 activation (C3c/native C3 ratio) was increased in subjects with highest lobular inflammation scores (**p<0.01). E) Gradually increasing hepatic properdin protein concentrations in subjects with higher lobular inflammation scores (*p<0.05). F) No correlation was observed between hepatic DAF protein levels and NAS score (r_s_ = 0.30; p = 0.34). G) Hepatic C3c protein levels were significantly correlated with NAS score in patients with NASH (r_s_ = 0.63; *p<0.05). H) A similar, but not significant correlation was observed between the C3c/native C3 activation ratio and NAS score (r_s_ = 0.51; p = 0.09). I) Hepatic Properdin protein levels did not correlated with NAS score (r_s_ = 0.42; p = 0.17).

Both properdin and C3adesArg, a C3 activation product, have been suggested to play a role in lipogenesis [15], [16]. In view of the accumulation of C3 activation products and properdin around steatotic hepatocytes that we observed, we investigated the potential association between hepatic C3c and properdin levels and the presence of steatosis. As shown above, hepatic C3c levels tended to be increased in subjects with steatosis (2.29±0.57 vs. 1.35±0.50, p = 0.07, see figure 2A). Properdin concentrations in the liver were similar in subjects with and without steatosis (3.73±0.83 vs. 2.72±0.25, p = 0.92, see figure 1A). In addition, higher grades of steatosis were not significantly associated with higher levels of C3c (grade 2: 1.45±0.49 vs. grade 3: 2.89±0.86, p = 0.27, figure 4A) or properdin (grade 2: 2.35±0.41 vs. grade 3: 4.71±1.31, p = 0.27, figure 4B). Thus, the extent of steatosis in NASH appears to be unrelated to alternative pathway induced generation of C3adesArg.

In contrast, we observed a gradual increase in C3c levels with a higher lobular inflammation score as determined by the Kleiner classification ((1) 0.73±0.11 vs. (2) 1.51±0.27 vs. (3) 4.43±1.00; p<0.01; figure 4C). A similar increase with higher lobular inflammation score was observed for the C3c/native C3 activation ratio ((1) 2.42±1.04 vs. (2) 2.27±0.44 vs. (3) 6.42±0.97; p<0.05; figure 4D). Remarkably, hepatic properdin protein concentrations also showed a similar significant increase with higher lobular inflammation scores ((1) 1.73±0.30 vs. (2) 2.75±0.59 vs. (3) 6.45±1.76; p<0.05, figure 4E). However, hepatic DAF protein levels were not associated with lobular inflammation ((1) 2.2±1.8 vs. (2) 1.1±0.9 vs. (3) 2.9±1.2, p = 0.20; data not shown). Similarly, DAF levels did not correlate with the NAFLD activity score (NAS) (r_s_ = 0.30; p = 0.34; figure 4F), whereas C3c levels did (r_s_ = 0.63; p<0.05; figure 4G). A similar trend was observed in the correlation of the C3c/native C3 activation ratio with the NAS score (r_s_ = 0.51, p = 0.09; figure 4H). Properdin protein levels (r_s_ = 0.42, p = 0.17; figure 4I) were not correlated to the NAS score. Taken together, these data suggest that hepatic inflammation in NASH is related to the generation of pro-inflammatory mediators by alternative pathway related C3 activation.

## Discussion

In the current study, we have shown that human NASH is characterized by reduced production but increased hepatic activation of C3, related to alternative pathway activation. Properdin, a positive regulator of the alternative pathway, co-localized with infiltrated neutrophils and the C3 activation product C3c, and correlated with hepatic C3c levels. Furthermore, both properdin and C3c levels gradually increased with lobular inflammation while factor H, an inhibitor of the alternative pathway, was downregulated in subjects with NASH. We provide the first evidence that activation of the alternative complement pathway and dysregulation of inhibitory factors of the alternative pathway are associated with hepatic inflammation in subjects with NASH.

Properdin is essential for activation of the alternative pathway of complement induced by bacterial endotoxin [17], a potent pro-inflammatory factor known to be involved in the pathogenesis of NASH [18]. Furthermore, properdin was recently shown to function as a pattern-recognition molecule which may serve in the identification and clearance of apoptotic cells [19]. Of note, we have previously reported an increased number of apoptotic cells in subjects with NASH which was associated with complement activation [5]. Therefore, properdin may promote the activation of the complement cascade at different levels in the liver of subjects with NASH.

Recently, elevated plasma levels of properdin and factor B were found in subjects at risk of developing type 2 diabetes [20]. This is consistent with a role for the alternative pathway in NASH, since insulin resistance is an independent risk factor for NASH [21]. Another key regulatory element of the alternative pathway of complement, factor H, has also been implicated in insulin resistance [22]. However, we found reduced factor H expression in the liver of subjects with NASH. Since factor H accelerates the decay of the alternative pathway C3 convertase, thereby restricting C3 activation, the observed low expression of factor H might underlie the increased hepatic C3c levels that we detected in the livers of subjects with NASH. Low factor H expression may also play a role in the detrimental effects of lipid peroxidation products in NASH [12], [23], since factor H was recently shown to recognize epitopes associated with oxidative stress and to mediate their neutralization [24].

Additional regulation of complement activation is performed by the action of DAF. Like factor H, DAF accelerates the decay of C3 convertases. In contrast to factor H, expression of DAF tended to be higher in subjects with NASH. This may be related to increased levels of TNF-alpha, IL-1beta, and IL-6, all of which have been shown to stimulate DAF expression [25], [26] and to be involved in the pathogenesis of NASH [27]. Furthermore, the terminal complement complex C5b9 is known to induce DAF [28], and we have previously shown that C5b9 is generated in the liver of humans with NASH [5]. Interestingly, hepatic DAF levels were positively correlated with C3 activation (C3c/C3 ratio), suggesting a feedback loop whereby C3 activation products promote downregulation of C3 convertase activity by DAF, limiting subsequent complement activation. Alternatively, increased DAF levels may function to compensate for the decreased factor H levels. In any case, the upregulation of DAF apparently could not prevent the activation of C3 as evident from the increased C3c levels in subjects with NASH. This indicates that the triggers of complement activation in human NASH are strong and persistent, and suggests that the downregulation of factor H combined with the local increase in properdin overwhelm the inhibitory effects of DAF.

Surprisingly, hepatic C3 mRNA and protein levels were strongly reduced in subjects with NASH. Hepatic C3 expression has been described to be increased in patients with cirrhotic-stage NASH [29]. More severe stages of NASH may therefore be accompanied by increased C3 synthesis. Furthermore, it has previously been shown that C3 plasma levels are higher in patients with NAFLD [30]. Type 2 diabetes, which is often present in patients with NASH, is also known to be associated with high systemic concentrations of C3 [31]. In view of the low C3 production in the NASH group that we observed, these high C3 levels may be attributable to extrahepatic synthesis of C3, e.g. by adipose tissue [32], [33]. Adipose tissue has also been described to produce C3a-desArg or acylation stimulating protein (ASP), a C3 derivative that stimulates triglyceride synthesis [16]. In fact, fasting ASP plasma levels have been described to be increased in human NAFLD [30], and ASP was therefore suggested to play a part in hepatic steatosis. Similarly, properdin was recently shown to be involved in lipid metabolism in mice [15]. In the current study, we did not find a significant association between the extent of lipid accumulation and the levels of the C3 activation product C3c or properdin, although there was a trend for increased C3c levels in subjects with steatosis.

In contrast, significant increases in both hepatic C3c and properdin levels were found in association with lobular inflammation, suggesting that the traditionally described function of the alternative pathway of complement, i.e. promoting inflammation, is important in human NASH. It is probable that complement-related pro-inflammatory products play a role in the pathogenesis of NASH. Neutrophils, an important component of inflammation in NASH [11], [12], are guided toward sites of complement activation by C3a and C5a, and stimulated to perform phagocytosis [34]. Hepatocyte cell death and ballooning, major hallmarks of NASH, can be induced by membrane attack complex formation [35], [36], which has been shown around steatotic hepatocytes [5]. C3a and C5a have also been shown to enhance Kupffer cell TNF-alpha expression [37], which appears to be crucial for the early phases of NASH development [38]. Thus, inhibition of the complement system may be a promising strategy for preventing the progression of NAFLD from the early, benign stages of simple steatosis, towards the more severe stages characterized by inflammation.

In summary, we have provided the first evidence that activation of the alternative pathway of complement occurs in human NASH, and that it is associated with its inflammatory component. Considering that the alternative pathway also amplifies complement activation due to the classical and lectin pathway activation that is known to occur in NASH, currently explored inhibitors of the alternative pathway such anti-factor B, anti-factor D, and the complement receptor 2/factor H fusion protein TT30 [6] could be attractive therapeutic agents for NASH.

## Supporting Information

Table S1 **Semi-quantitative analysis of NASH severity in the NASH group according to the Brunt and Kleiner classification.**
(DOC)

Table S2 **Gene-specific primers used for quantitative polymerase chain reaction analysis.**
(DOC)
