# Supplementary material for: Complement Alternative Pathway Activation in Human Nonalcoholic Steatohepatitis
Source: PLoS One. 2014 Oct 9;9(10):e110053. doi: 10.1371/journal.pone.0110053 (PMC4192551; doi:10.1371/journal.pone.0110053)
Supplement: Table S1 — Semi-quantitative analysis of NASH severity in the NASH group according to the Brunt and Kleiner classification. (DOC) [file pone.0110053.s001.doc]

Supplemental Table 1:

|  | **Score** | **Number of subjects** |
| --- | --- | --- |
| **Brunt grade** | 1  2  3 | 2  6  4 |
| **Brunt stage** | 0  1  2  3 | 1  3  3  5 |
| **Kleiner steatosis grade** | 0  1  2  3 | 0  0  5  7 |
| **Kleiner lobular inflammation** | 1  2  3 | 3  5  4 |
| **Kleiner ballooning** | 0  1  2 | 0  9  3 |
| **Kleiner fibrosis** | 0  1  2  3  4 | 1  3  3  4  1 |
| **Kleiner NAS score** | 6  7  8  9  10  11  12 | 1  5  3  1  1  0  1 |
