# Supplementary material for: Complement Alternative Pathway Activation in Human Nonalcoholic Steatohepatitis
Source: PLoS One. 2014 Oct 9;9(10):e110053. doi: 10.1371/journal.pone.0110053 (PMC4192551; doi:10.1371/journal.pone.0110053)
Supplement: Table S2 — Gene-specific primers used for quantitative polymerase chain reaction analysis. (DOC) [file pone.0110053.s002.doc]

Supplemental Table 2:

| Gene |  | Sequence primer (5' - 3') |
| --- | --- | --- |
| Factor B | Fw | GGCAGGCCAAGATCTCAGTCATTC |
|  | Rev | CACAAAGTACTCAGACACCACAGC |
| Factor D | Fw | AGGGTCACCCAAGCAACAAAGTC |
|  | Rev | ACCAACCAGATGCAGGAGTGGATG |
| Factor H | Fw | ATGTACCTTGAAACCTTGTG |
|  | Rev | AATGTGATCCCAGTAACTTCC |
| DAF | Fw | TTTGTCTTATTTCAGGCAGCTC |
|  | Rev | CTCCAATCATGGTGAATCCT |
| C3 | Fw | GCTGTCAGCATGTCGGACAA |
|  | Rev | CCTGCATTACTGTGACCTCGAA |
| Cyclophylin A | Fw | CTCGAATAAGTTTGACTTGTGTTT |
|  | Rev | CTAGGCATGGGAGGGAACA |
| B2-microglobulin | Fw | TCCATCCGACATTGAAGTTG |
|  | Rev | CGGCAGGCATACTCATCTT |
